# A Deep Learning-Based Graphical User Interface for Predicting Corneal Ectasia Scores from Raw Optical Coherence Tomography Data

**DOI:** 10.3390/diagnostics16020310

**Published:** 2026-01-18

**Authors:** Maziar Mirsalehi, Achim Langenbucher

**Affiliations:** Department of Experimental Ophthalmology, Saarland University, Kirrberger Street 100, 66424 Homburg, Germany

**Keywords:** CNN, cornea, deep learning, ectasia, eye, keratoconus, OCT, raw data, vision

## Abstract

**Background/Objectives**: Keratoconus, a condition in which the cornea becomes thinner and steeper, can cause visual problems, particularly when it is progressive. Early diagnosis is important for preserving visual acuity. Raw data, unlike preprocessed data, are unaffected by software modifications. They retain their native structure across versions, providing consistency for analytical purposes. The objective of this study was to design a deep learning-based graphical user interface for predicting the corneal ectasia score using raw optical coherence tomography data. **Methods**: The graphical user interface was developed using Tkinter, a Python library for building graphical user interfaces. The user is allowed to select raw data from the cornea/anterior segment optical coherence tomography Casia2, which is generated in the 3dv format, from the local system. To view the predicted corneal ectasia score, the user must determine whether the selected 3dv file corresponds to the left or right eye. Extracted optical coherence tomography images are cropped, resized to 224 × 224 pixels and processed by the modified EfficientNet-B0 convolutional neural network to predict the corneal ectasia score. The predicted corneal ectasia score value is displayed along with a diagnosis: ‘No detectable ectasia pattern’ or ‘Suspected ectasia’ or ‘Clinical ectasia’. Performance metric values were rounded to four decimal places, and the mean absolute error value was rounded to two decimal places. **Results**: The modified EfficientNet-B0 obtained a mean absolute error of 6.65 when evaluated on the test dataset. For the two-class classification, it achieved an accuracy of 87.96%, a sensitivity of 82.41%, a specificity of 96.69%, a positive predictive value of 97.52% and an F1 score of 89.33%. For the three-class classification, it attained a weighted-average F1 score of 84.95% and an overall accuracy of 84.75%. **Conclusions**: The graphical user interface outputs numerical ectasia scores, which improves other categorical labels. The graphical user interface enables consistent diagnostics, regardless of software updates, by using raw data from the Casia2. The successful use of raw optical coherence tomography data indicates the potential for raw optical coherence tomography data to be used, rather than preprocessed optical coherence tomography data, for diagnosing keratoconus.

## 1. Introduction

The eye is an important part of the human body. It contains tissues such as the cornea, sclera, lens, iris, vitreous, aqueous humour, retina and optic nerve. The cornea has two-thirds of the refractive power of the eye, which makes it important for vision. The cornea has five layers, including the epithelium, stroma and endothelium as cellular layers and Bowman’s layer and Descemet’s membrane as acellular layers [1,2].

Keratoconus is a condition in which the cornea becomes thinner and steeper. It commonly impacts both eyes, although the severity can vary. This condition can lead to vision problems and can be found in both males and females, with approximately 1 in 2000 people experiencing it. However, it is predominant in males, starting mostly in the second or, at the latest, the third decade of life. Eye rubbing is considered a major risk factor for keratoconus. Early keratoconus can be identified using various diagnostic tools, including handheld keratoscopes (Placido discs), slit-lamp biomicroscopy, ultrasonic pachymetry and techniques such as corneal topography and tomography, like Scheimpflug imaging and Optical Coherence Tomography (OCT). Scheimpflug imaging uses a rotating camera to generate images, while OCT measures the time delay of reflected light from the cornea’s anterior surface by relating it to a reference. Corneal topography illustrates the form of the cornea’s anterior surface, whereas corneal tomography provides a three-dimensional representation of the entire cornea. Placido-based topography primarily assesses the central anterior corneal surface and may fail to detect posterior elevation abnormalities, which are indicative of early keratoconus. Disadvantages of slit-lamp biomicroscopy include a dependence on trained professionals and difficulty in detecting subtle early signs of keratoconus. A disadvantage of ultrasonic pachymetry is the possibility of corneal damage due to repeated contact with the probe. Corneal topographers have limitations, such as a sensitivity to non-optimal conditions, like dry eye syndrome and long eyelashes; poor repeatability in children; high costs; and limited portability. Corneal tomography is costly, not portable, requires clinical expertise and depends on patient cooperation, which limits its use in large-scale screening and makes it impractical in many regions. OCT involves an expensive, non-portable tool that requires technician training. Its limited effectiveness when used alone for detecting early keratoconus emphasises the need for complementary imaging techniques or Artificial Intelligence (AI)-based methods to improve accuracy [3,4,5,6,7,8,9].

High-resolution anterior segment tomographers are excellent tools to image the cornea and the entire anterior eye segment, and most of them have proprietary (undisclosed) software tools that assist in screening or diagnosing ectatic diseases such as keratoconus. The data can typically be used in its raw form or preprocessed form. Preprocessed data is modified by software, and the changes made may not be completely clear. Additionally, software updates may introduce variations in data processing that cause inconsistencies in the results. Unlike processed data, raw data is not modified by software. Consequently, it maintains its original structure across various software versions, providing a more consistent basis for analysis. According to our knowledge, this is the first study to apply raw OCT data in a Graphical User Interface (GUI) for the prediction of corneal ectasia scores. The GUI outputs numerical ectasia scores, improving over categorical labels, and ensures consistent results from three-dimensional optical coherence tomography data, unaffected by software changes.

## 2. Materials and Methods

### 2.1. Data

Data were collected from patients examined at the Department of Ophthalmology, Saarland University Medical Centre, Homburg, Germany, between 1 February 2021 and 1 September 2023 using the cornea/anterior segment OCT Casia2 (Tomey, Nagoya, Japan) for data acquisition. Casia2 is an OCT-based diagnostic instrument. Raw data generated by the Casia2 system are stored in 3dv format, with individual files corresponding to corneal maps. Every 3dv file consists of data from 16 equiangular meridional images. These images were stored as 16-bit unsigned integers. The 16 equiangular meridional images from each 3dv file—each with a resolution of 800 pixels in width and 1464 pixels in height, with a horizontal measurement range of 16 mm and a depth of 11 mm in anterior segment mode—were first extracted and then saved as greyscale Portable Network Graphic (PNG) files. The Ectasia Screening Index (ESI) is a value provided by the Casia2 for screening corneal ectasia. If the ESI result is within the range of 0 to 4, no ectasia pattern is detectable. A value ranging from 5 to 29 indicates suspected ectasia, whereas a value ranging from 30 to 95 indicates clinical ectasia. The ESI is recorded for each measurement in a CSV (Comma-Separated Value) file; 6141 files from 15,457 were chosen for training, validation and testing. The remaining files were disregarded due to corneal defects like keratoplasty. The data were split into three separate sets, including 4898 files for training, 620 files for validation and 623 files for testing.

Two classification approaches were evaluated: a two-class classification separating the no detectable ectasia pattern from suspected ectasia and clinical ectasia combined into a single class and a three-class classification distinguishing the no detectable ectasia pattern, suspected ectasia and clinical ectasia. For the two-class classification, files were categorised according to an ESI threshold of 5, as defined by the Casia2. Files with an ESI below 5 are categorised as NE (No Ectasia), indicating no detectable ectasia pattern, whereas files with an ESI of 5 or higher are categorised as E (Ectasia), indicating either suspected ectasia or clinical ectasia. In the training set, 2065 files belonged to the NE class, and 2833 files belonged to the E class. In the validation set, 242 files were classified as NE, and 378 files were classified as E. For the test set, 242 files belonged to the NE class, and 381 files belonged to the E class. Table 1 summarises the distribution of data for the two-class classification across the training, validation and test sets.

For the three-class classification, files with an ESI below 5, according to the Casia2 criteria, are categorised as NE (No Ectasia), which indicates no detectable ectasia pattern. Files with an ESI ranging from 5 to 29, according to the Casia2 criteria, are categorised as SE (Suspected Ectasia), indicating suspected ectasia, while files with an ESI ranging from 30 to 95, according to the Casia2 criteria, are categorised as CE (Clinical Ectasia), which indicates clinical ectasia. In the training set, 2065 files belong to the NE class, 907 files belong to the SE class and 1926 files belong to the CE class. In the validation set, 242 files are classified as NE, 117 files belong to the SE class and 261 files belong to the CE class. For the test set, 242 files belong to the NE class, 103 files belong to the SE class and 278 files belong to the CE class. Table 2 summarises the distribution of data for the three-class classification across the training, validation and test sets.

In this study, age and sex were not regarded as critical factors.

**Figure 1:**
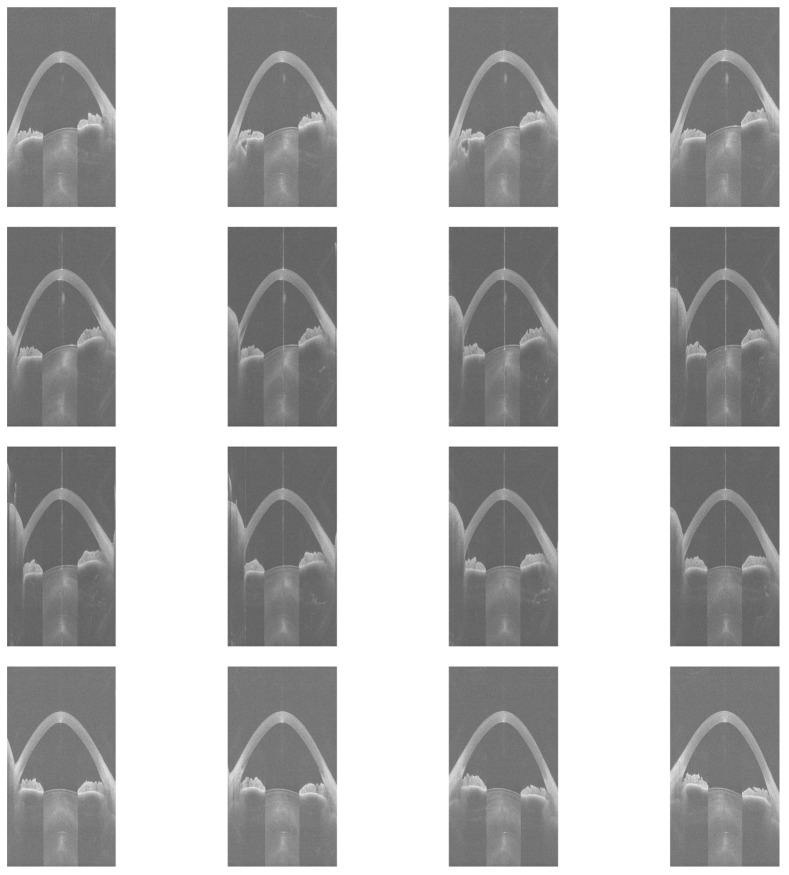
Sixteen equiangular meridional images extracted from a 3dv file.

Figure 1 shows 16 equiangular meridional images extracted from a 3dv file, which corresponds to a right eye examination with an ESI of 47.

**Figure 2:**
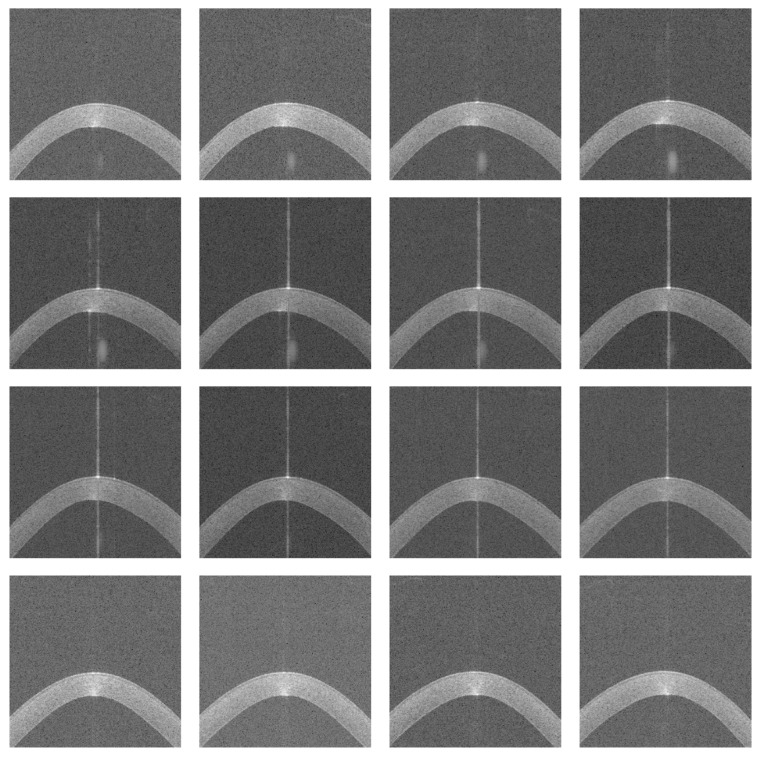
Cropped and resized images.

The image preprocessing consisted of trimming 25% from the left edge and 25% from the right edge of the images to remove irrelevant eyelid portions, as well as cropping 60% from the bottom to exclude areas outside the cornea. Following this, the images were resized to 224 × 224 pixels, which matches the initial resolution chosen for the EfficientNet-B0 model [10]. Figure 2 illustrates the cropped and resized versions of the images extracted from the same 3dv file shown in Figure 1.

### 2.2. Training Architecture

EfficientNet-B0 [10] was employed as the CNN architecture in the GUI. Python (version 3.12.3) [11] and the PyTorch library (version 2.3.0) [12] were used to train the architecture from scratch. Training was conducted on a system with an 11th Gen Intel^®^ Core™ i7-11700 processor running at 2.5 GHz, supported by 32 GB of RAM and equipped with a 64-bit operating system and an x64-based processor.

Each set of 16 cropped and resized images was combined into a single stacked input and provided to the EfficientNet-B0 model. The initial convolutional layer of EfficientNet-B0 was modified to accept a 16-channel input. The classifier head was adapted for a single-output task and consists of two fully connected layers with dimensions of 1280 to 1000 and 1000 to 1, with a dropout layer applied to the output of the first fully connected layer, with a dropout probability of 0.2 to reduce overfitting. An additional fully connected fusion layer was incorporated to combine the single output from the classifier with two eye parameter features corresponding to the left and right eyes. This fusion layer has three input features and one output feature and employs a linear (identity) activation function. The additional fully connected layer produces a single numerical value as the CNN architecture’s final output. The total number of trainable parameters in the proposed model is 5,293,297.

Table 3 shows the differences between the original EfficientNet-B0 and the modified version used in this study.

**Figure 3:**
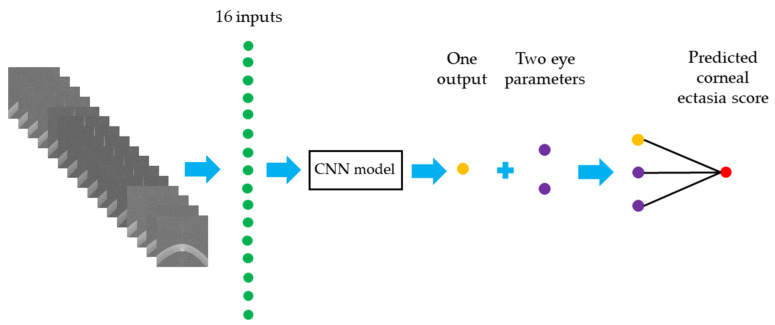
A workflow diagram for predicting the corneal ectasia score. Abbreviation: CNN = Convolutional Neural Network.

The architecture was trained for 80 epochs to ensure sufficient learning. The batch size for the training, validation and test sets was 128. The loss function chosen to minimise prediction errors was the Mean Squared Error (MSE). To optimise the architecture parameters, the AdamW optimiser was used with a learning rate of 0.01, as [13] found that AdamW results in faster convergence and better generalisation compared to Adam. A learning rate of 0.01 is considered effective because it offers a balance between being large enough for faster convergence and small enough to avoid instability or overshooting the optimal solution, ensuring efficient training. A learning rate scheduler was also applied to change the learning rate when a plateau was reached, with a reduction factor of 0.1 and a patience of 5 epochs. Specifically, the scheduler monitors the validation loss and reduces the learning rate by a factor of 0.1 if no improvement is observed for 5 consecutive epochs. Additionally, a weight decay of 0.04 was applied to help prevent overfitting by regularising the architecture’s parameters. Figure 3 illustrates the workflow for predicting the corneal ectasia score by using the modified EfficientNet-B0.

### 2.3. Evaluation Metrics

The Receiver Operating Characteristic (ROC) curve was examined to determine the ideal balance between sensitivity and specificity for the predictions by identifying the optimal threshold, which is the point that maximises the difference between sensitivity and (1- specificity). Corneal ectasia scores below the threshold were classified as NE, indicating no detectable ectasia pattern, while scores at or above the threshold were categorised as E, indicating either suspected ectasia or clinical ectasia. Based on the True Positive (TP), False Positive (FP), True Negative (TN) and False Negative (FN) values for both categories, commonly used performance metrics were applied to assess the classification effectiveness of the modified EfficientNet-B0 architecture. These metrics, including accuracy, sensitivity, specificity, the Positive Predictive Value (PPV) and the F1 score, were computed following the definitions provided in [14]. Performance metric values and the area under the curve were rounded to four decimal places. The Youden index was rounded to two decimal places.

The Mean Absolute Error (MAE) was also used as a performance metric to assess how much, on average, the predicted corneal ectasia score values deviate from the actual ESI values. The MAE is defined as the average absolute difference between predicted and actual values, as described in [15]. The MAE value was rounded to two decimal places.

### 2.4. Gradient-Weighted Class Activation Mapping

Gradient-Weighted Class Activation Mapping (Grad-CAM) [16] was used to visualise the areas within the CNN architecture that most strongly influenced the prediction of the corneal ectasia score in the test dataset. A Python script was developed to run the model and generate Grad-CAM outputs for the test data. For the CNN architecture, Grad-CAM was applied to the final convolutional layer.

### 2.5. The Implementation of the Graphical User Interface

The GUI was created using Tkinter (version 8.6), a Python library for constructing GUIs. For image processing tasks, such as reading, cropping and resizing, OpenCV (cv2) (version 4.12.0) was used. The modified EfficientNet-B0 architecture was implemented by using PyTorch (version 2.7.1). The trained architecture was saved for subsequent use in the GUI. Matplotlib (version 3.10.3) was used for the visualisation and saving of images, and NumPy (version 2.2.6) was used for numerical operations on image data. Pathlib was employed for managing file paths. The GUI was developed on a system running Python (version 3.13.5). The predicted corneal ectasia score value was rounded to two decimal places.

## 3. Results

**Figure 4:**
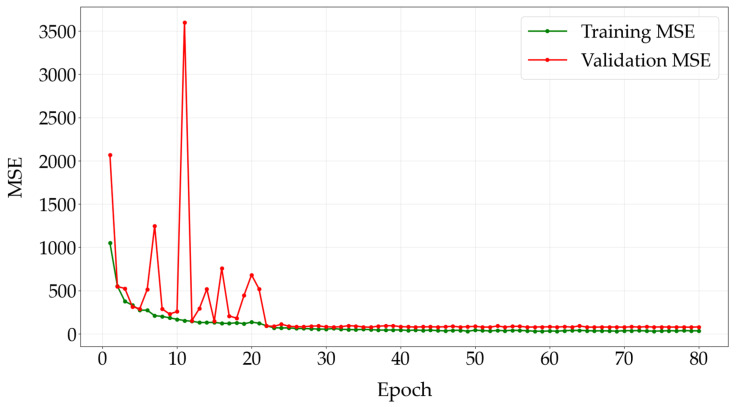
Training and validation Mean Squared Error across epochs. Abbreviation: MSE = Mean Squared Error.

Figure 4 shows the progression of the training and validation MSE over the epochs for the modified EfficientNet-B0 architecture.

**Figure 5:**
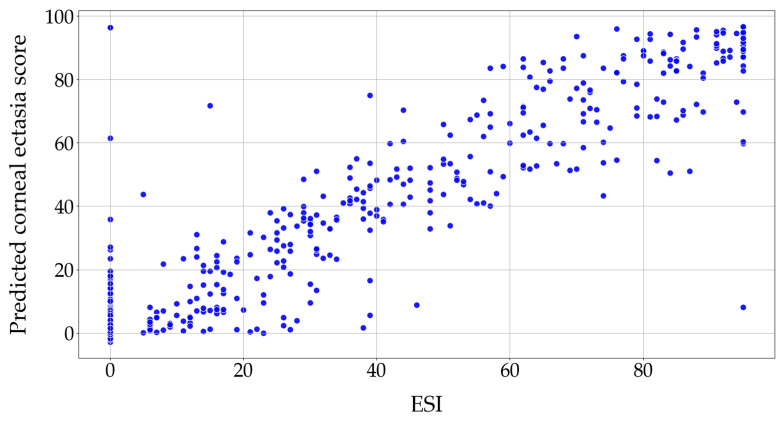
The correlation between the actual ESIs and the modified EfficientNet-B0 predictions. Abbreviation: ESI= Ectasia Screening Index.

Figure 5 shows the correlation between the actual ESIs and the corneal ectasia scores predicted by the modified EfficientNet-B0.

**Figure 6:**
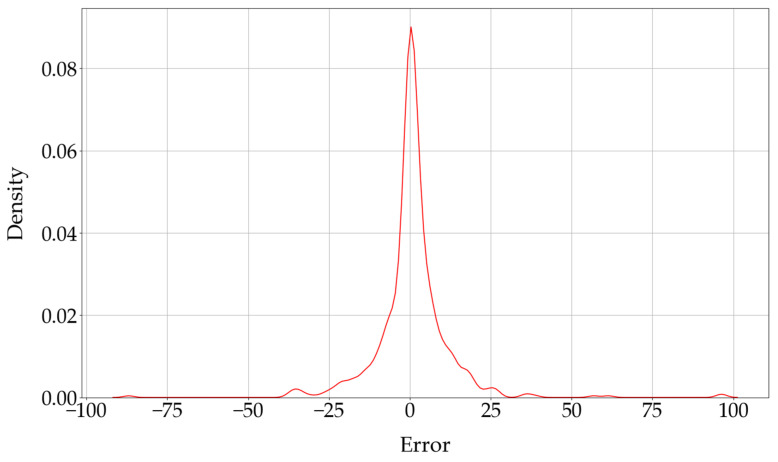
The KDE of the errors between the actual ESIs and the corneal ectasia scores predicted by the modified EfficientNet-B0. Abbreviation: KDE = Kernel Density Estimate.

Figure 6 shows the Kernel Density Estimate (KDE) of the errors between the actual ESIs and the corneal ectasia scores predicted by the modified EfficientNet-B0. This KDE plot represents the distribution of errors, where the error is determined by subtracting the actual ESI from the predicted corneal ectasia score.

**Figure 7:**
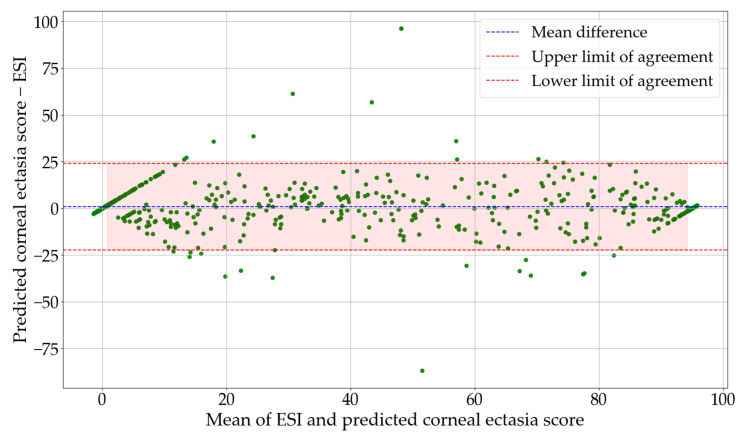
Bland–-Altman plot of ESI values and predicted corneal ectasia scores for the test dataset. Abbreviation: ESI = Ectasia Screening Index.

The Bland–Altman plot illustrates the agreement between the ESI values and the predicted corneal ectasia scores. The mean difference was 0.7831, which shows a small positive bias in the predictions. The 95% confidence interval for the mean difference ranged from −0.1463 to 1.7125. The standard deviation of the differences was 11.8353, which describes the variability between the predicted corneal ectasia scores and the ESI values. The lower and upper limits of agreement were −22.4140 and 23.9802, respectively, with corresponding 95% confidence intervals of −45.6298 to 0.8017 for the lower limit and 0.7645 to 47.1960 for the upper limit. Figure 7 shows the Bland–Altman plot of ESI values versus predicted corneal ectasia scores for the test dataset.

**Figure 8:**
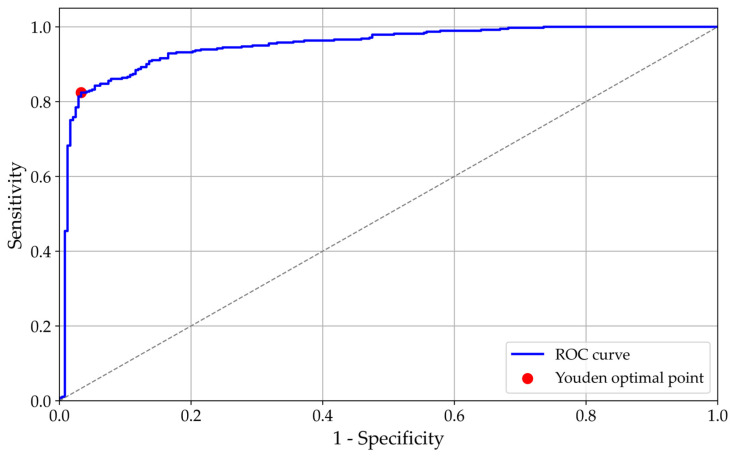
ROC curve. Abbreviation: ROC = Receiver Operating Characteristic.

Figure 8 shows the ROC curve for the architecture. The area under the curve is 0.9496, and the optimal threshold, determined using the Youden index, is 18.53. The optimal threshold was determined by computing the Youden index for each threshold in the ROC curve and selecting the threshold that maximised the index (Youden index = sensitivity − (1 − specificity)).

**Figure 9:**
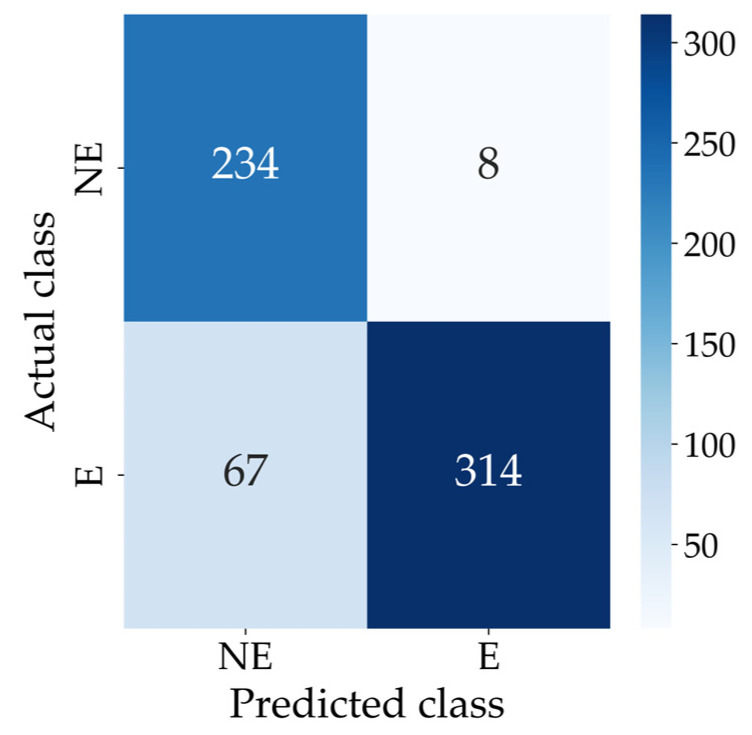
Confusion matrix for the two-class classification. Abbreviations: E = Ectasia and NE = No Ectasia.

Figure 9 shows the confusion matrix for the two-class classification.

For the two-class classification, the modified EfficientNet-B0 achieved an accuracy of 0.8796, a sensitivity of 0.8241, a specificity of 0.9669, a PPV of 0.9752 and an F1 score of 0.8933. Also, the architecture achieved an MAE of 6.65 when evaluated on the test dataset. Table 4 summarises the performance of the modified EfficientNet-B0 for the two-class classification on the test dataset.

The optimal thresholds for classifying corneal ectasia were approximately 4.51 and 31.85, both rounded to two decimal places. These thresholds were obtained by performing a grid search of 500 steps across the range of predicted corneal ectasia scores. For each candidate pair of thresholds (t1, t2) with t1 < t2, predictions were generated by assigning classes according to the thresholds, and the resulting weighted F1 score was calculated against the ground truth labels, which were derived from the ESI values. The thresholds that maximised the weighted F1 score were selected as optimal, ensuring the best balance between the PPV and sensitivity for the NE, SE and E classes.

**Figure 10:**
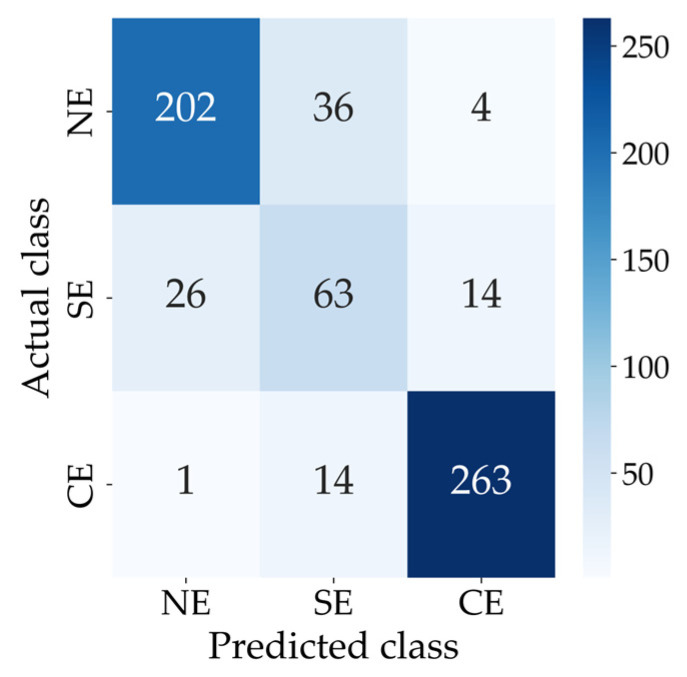
Confusion matrix for the three-class classification. Abbreviations: CE = Clinical Ectasia, NE = No Ectasia and SE = Suspected Ectasia.

Figure 10 shows the confusion matrix for the three-class classification.

Table 5 summarises the performance metrics of the modified EfficientNet-B0 for the three-class classification on the test dataset. The sensitivity, specificity, PPV, F1 score and overall accuracy are reported for each class (NE, SE and E), along with the macro- and weighted averages across all classes. The model achieved a weighted-average F1 score of 0.8495 and an overall accuracy of 0.8475.

**Figure 11:**
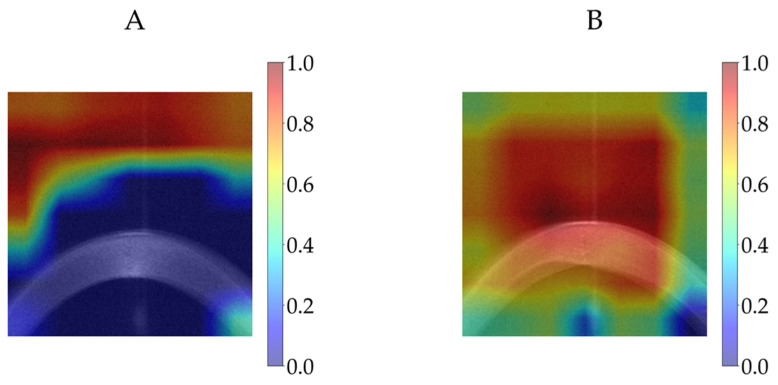
Grad-CAM visualisations for two eye examinations using the modified EfficientNet-B0 on the test dataset: (**A**) a predicted corneal ectasia score of 3.11 for an ESI value of 0 and (**B**) a predicted corneal ectasia score of 95.04 for an ESI value of 95 (both predicted corneal ectasia scores are rounded to two decimal places). Abbreviations: ESI = Ectasia Screening Index and Grad-CAM = Gradient-Weighted Class Activation Mapping.

Figure 11 shows the Grad-CAM visualisations for two eye examinations produced by the modified EfficientNet-B0 architecture on the test dataset: (A) the predicted corneal ectasia score was 3.11 (rounded to two decimal places) for an ESI value of 0, and (B) the predicted corneal ectasia score was 95.04 (rounded to two decimal places) for an ESI value of 95. The relative importance of the CNN’s decisions is shown with colour coding, which ranges from blue, to indicate little or no influence, to red, to indicate a strong influence on the prediction. The Grad-CAM corresponding to each prediction was overlaid onto the average of all sixteen resized and extracted images.

As shown in Figure S1, the GUI features a light background and three buttons, labelled ‘File,’ ‘Images’ and ‘Prediction,’ arranged horizontally in the centre of the screen.

When the user clicks the ‘File’ button, a window appears, allowing the selection of a 3dv file from the local system, as shown in Figure S2.

The application then checks the file to confirm it is the correct type (3dv) and has the required size of 36,600 KB. If the file is valid, a window appears displaying the message ‘The selected file is valid’. If the file is not in 3dv format or does not meet the size requirement, the user receives an error message stating ‘The selected file is not valid. The file must be a 3dv file and have a size of 36,600 KB’. Once a valid 3dv file is selected, the ‘Images’ button allows the user to view the extracted OCT images. When the button is clicked, the system processes the data from the selected file, reshapes it into 16 separate OCT images and arranges them in a 4 × 4 grid. The user can save the figure and zoom in on any of the 16 individual images within the grid.

Figure S3 shows the display of 16 OCT images from the selected file, 614711-19086-1.3dv, which belongs to the left eye. The user can click the ‘Prediction’ button to begin the corneal ectasia score prediction process.

Once this button is clicked, a window appears with the message ‘Enter r for right eye or l for left eye,’ as shown in Figure S4. The user is required to specify whether the selected 3dv file corresponds to the left or right eye by entering ‘l’ for the left eye or ‘r’ for the right eye. The eye side is a critical input, as it affects the accuracy of the analysis.

Once the input is provided, the application requests the user to select a directory in which to save the images extracted from the selected file. The user must choose a location to store the extracted images from the selected 3dv file. After saving the images, the application processes the OCT images by cropping and resizing them. These processed images are subsequently input into the modified EfficientNet-B0 architecture to predict the corneal ectasia score. The predicted corneal ectasia score is displayed to the user, along with a classification indicating whether the class is labelled as NE or SE or CE. If the class is NE, the application displays ‘Diagnosis: No detectable ectasia pattern’. If the class is SE, it shows ‘Diagnosis: Suspected ectasia’. If the class is CE, it displays ‘Diagnosis: Clinical ectasia’. As shown in Figure S5, the predicted corneal ectasia score for the selected 3dv file is 54.42, which indicates ectasia, while the actual ESI is 51, which also suggests clinical ectasia based on the Casia2 threshold, where an ESI value of 30 to 95 indicates clinical ectasia.

## 4. Discussion

This method, which uses a user-friendly GUI, predicts corneal ectasia as a numerical score. On average, the predictions differ from the ESI values by 6.65, as measured by the MAE. With the numerical corneal ectasia prediction provided by the GUI, users can track and compare differences in corneal ectasia scores across multiple measurements, which allows them to observe trends in the condition’s progression relative to the established threshold for a sensitive and dynamic assessment. The F1 score is particularly valuable when working with imbalanced datasets, where one class greatly outweighs the other. It serves as a more dependable metric for evaluating the architecture performance in such cases. Traditional measures like accuracy, sensitivity and specificity may not fully capture how effectively the architecture differentiates between classes. In contrast, the F1 score offers a more comprehensive assessment by factoring in both the sensitivity and PPV. Although the validation MSE fluctuated in the early epochs, its overall trend was downward, and it converged by epoch 80, which indicates that the architecture successfully learned the data without overfitting.

Ruiwei Feng et al. [17] developed KerNet, a deep learning model for detecting keratoconus and subclinical keratoconus based on a dataset that included 854 raw data samples from the Pentacam HR system. The model achieved a 94.74% accuracy, 93.71% sensitivity, 94.10% PPV and 93.89% F1 score. Jan Schatteburg et al. [18] developed a method for diagnosing keratoconus using Convolutional Neural Networks (CNNs) based on the ESI, with data collected using the SS-1000 Casia OCT Imaging System. However, the study lacked evaluation metrics. Benjamin Fassbind et al. [19] investigated keratoconus detection by using the CorNeXt CNN model, which builds on the ConvNeXt architecture [20], with topography maps generated from the Casia2 anterior OCT. The model achieved a 92.56% accuracy, 84.07% sensitivity, 100% specificity and a 91.34% F1 score for keratoconus detection. PeiPei Zhang et al. [21] applied the CorNet model to diagnose keratoconus by using raw data samples from the Corvis ST, a non-contact device that assesses corneal biomechanics through air-puff-induced deformation. The model achieved a 92.13% accuracy, 92.49% sensitivity, 91.54% specificity, a 94.77% PPV and a 93.62% F1 score. Hazem Abdelmotaal et al. [22] developed a DenseNet121-based CNN model to distinguish between normal eyes and those with keratoconus. The study utilised 734 video clips from the ST Corvis machines, each representing a different eye. Two datasets were employed. One comprised 502 subjects, including 259 normal eyes and 243 with keratoconus, and was randomly divided into 70% for training and validation and 30% for testing. The model achieved an accuracy of 89% on the test set of this dataset. Another dataset consisted of 232 subjects, including 131 normal eyes and 101 with keratoconus, which was used as the external validation set. The model reached an accuracy of 88% on this dataset. Wiyada Quanchareonsap et al. [23] tested three AI models based on EfficientNet-B7 to distinguish between normal corneas, subclinical keratoconus and keratoconus by using Pentacam tomographic maps and Corvis ST corneal biomechanics data. AI model 1, which used only Pentacam data, achieved an accuracy of 94.7%, a sensitivity of 90.8%, a specificity of 96.9% and a PPV of 92.4%. AI model 2 attained an accuracy of 95.6%, sensitivity of 93.0%, specificity of 94.3% and PPV of 92.9% by combining Corvis ST data with AI model 1. AI model 3 obtained an accuracy of 95.6%, sensitivity of 93.0%, specificity of 94.3% and PPV of 92.9% by incorporating the corneal biomechanical index into AI model 2. I. Kallel Fourati et al. [24] proposed a Matlab-based GUI for the monitoring and early detection of keratoconus. The study achieved accuracies of 96% and 92% for binary and three-class classifications, respectively. The binary classification separated normal corneas and keratoconus by using ten parameters from ORBSCAN images. The three-class system included normal eyes, suspected keratoconus and keratoconus, based on ORBSCAN clinical features. Additionally, combining ORBSCAN features with OCT-derived corneal pachymetry measures and clinical judgement improved the accuracy to 99% for two classes and 94% for three classes. The dataset comprised 98 patient records, each including ORBSCAN and OCT images. An artificial neural network with 10 input neurons, a hidden layer of 11 neurons and an output layer was used. Despite a high reported accuracy, metrics such as sensitivity, specificity, PPVs and F1 scores were not reported. The small size of the dataset affects the extent to which the results can be generalised. Alexandru Lavric et al. [25] applied 25 machine learning algorithms in Matlab to detect keratoconus using real-world data from 3151 corneal images of 3146 eyes, collected with SS-1000 CASIA OCT systems across several clinics in Japan. Each sample included 443 corneal parameters, and the ESI was used as the ground truth, classifying eyes as normal (ESI between 0 and 4), suspect or forme fruste keratoconus (ESI between 5 and 29) or keratoconus (ESI equal to or greater than 30). The dataset comprised 1970 normal eyes, 791 eyes with forme fruste keratoconus and 390 eyes with keratoconus. Two classification schemes were evaluated: a binary scheme separating normal from suspect or keratoconus eyes and a three-class scheme identifying normal eyes, suspect keratoconus and keratoconus eyes. The models achieved an accuracy range of 62% to 94%, with 3003 samples used for training and testing and 148 samples used for final validation.

Based on the results of this study, the GUI’s optimal thresholds of 4.51 and 31.85, both rounded to two decimal places, correspond closely to the Casia2 thresholds of 5 and 30, respectively. This suggests that the GUI’s predicted thresholds align closely with the Casia2 system in discriminating between no detectable ectasia pattern, suspected ectasia and clinical ectasia cases, while using raw OCT data that are independent of Casia2 software updates. This makes it more reliable for comparing corneal ectasia scores across different OCT-based devices, regardless of software updates or processing variations such as noise removal. The user has the option to view the 16 extracted images from the raw 3dv file and zoom in on each image. This feature allows the user to compare the diagnosis provided by the GUI with what they observe in the images.

However, this study has six limitations, which should be taken into consideration. First, of the 15,457 files, 9316 were excluded, which resulted in 6141 images being used for model development. Exclusions were primarily due to movement artefacts, post-Penetrating Keratoplasty (PK) cases and narrow lid openings. Although exact counts for each criterion were not recorded individually, these exclusions may have influenced the dataset’s distribution by under-representing certain anatomical variations and post-surgical cases. Consequently, the model’s performance may be less generalisable to images with movement artefacts, post-PK eyes or cases with incomplete eyelid exposure. Future work should include these under-represented cases to evaluate and potentially improve external validity. Second, only one CNN model was modified and tested, and it is possible that other more advanced models could yield more accurate or robust predictions. Third, the eye side is a critical input, as it affects the accuracy of the analysis, and further research could remove its significance in the analysis. Fourth, this study did not independently validate the Casia2-generated ESI scores against a gold standard. Consequently, potential discrepancies between the ESI and clinical diagnosis may exist. Fifth, the architecture was trained to mimic the ESI rather than a clinical ground truth. Consequently, the proposed score should be interpreted as a proxy for the ESI, and its direct clinical relevance requires validation against an expert clinical diagnosis in future studies. Sixth, the proposed score was evaluated only on Casia2 OCT data from a single institution, which limits the assessment of its generalisability across different devices or clinical settings. While the model reproduces the Casia2-generated ESI accurately within our dataset, its performance on data from other devices or institutions remains to be established. Future studies should evaluate the score on independent datasets to confirm its robustness and broader applicability. These limitations highlight areas for further investigation and improvement, which could enhance the overall accuracy and applicability of the GUI.

## 5. Conclusions

The GUI uses raw 3dv files from the Casia2 device as input for predicting the corneal ectasia score and for classifying the condition of the cornea into three classes of no detectable ectasia pattern, suspected ectasia and clinical ectasia. Using raw data guarantees reliable diagnostics that remain unaffected by software modifications. This strategy has the advantage that, unlike using preprocessed data for predicting the condition of the cornea, it eliminates the influence of factors such as noise removal and filtering. By using raw data, one can be certain that such processes do not affect the prediction results. Therefore, if different devices use different software but follow the same method for capturing images (in this case, OCT), this approach provides a fair strategy for comparing prediction results across different models. Moreover, saving the extracted images through the GUI enables the user to compare their own assessment of the corneal condition (no detectable ectasia pattern, suspected ectasia and clinical ectasia) with the GUI’s prediction. This gives the user another opportunity to independently determine the condition of the cornea and make a more reliable decision in case the GUI misclassifies it. This GUI also produces a numerical corneal ectasia score prediction, which enables the user to compare the degree of ectasia across multiple measurements.

## Figures and Tables

**Table 1:** The distribution of the dataset for the two-class classification across training, validation and test sets. Abbreviations: NE = No Ectasia and E = Ectasia.

Dataset	NE	E	Total
Training	2065	2833	4898
Validation	242	378	620
Test	242	381	623

**Table 2:** The distribution of the dataset for the three-class classification across training, validation and test sets. Abbreviations: NE = No Ectasia, SE = Suspected Ectasia and CE = Clinical Ectasia.

Dataset	NE	SE	CE	Total
Training	2065	907	1926	4898
Validation	242	117	261	620
Test	242	103	278	623

**Table 3:** A comparison of the original CNN models with their modified versions. Abbreviation: CNN = Convolutional Neural Network.

CNN Model	Number of Input Channels	Number of Output Features
EfficientNet-B0	3	1000
Modified EfficientNet-B0	16	1

**Table 4:** Performance metrics of the modified EfficientNet-B0 for the two-class classification, tested on the test dataset. Abbreviation: PPV = Positive Predictive Value.

Sensitivity	Specificity	PPV	F1 Score	Accuracy
0.8241	0.9669	0.9752	0.8933	0.8796

**Table 5:** Performance metrics of the modified EfficientNet-B0 for the three-class classification, tested on the test dataset. Abbreviations: CE = Clinical Ectasia, NE = No Ectasia, PPV = Positive Predictive Value and SE = Suspected Ectasia.

Group	Sensitivity	Specificity	PPV	F1 Score	Overall Accuracy
NE	0.8347	0.9291	0.8821	0.8577	0.8475
SE	0.6117	0.9038	0.5575	0.5833	
CE	0.9460	0.9478	0.9359	0.9410	
Macro average	0.7975	0.9269	0.7919	0.7940	
Weighted average	0.8475	0.9333	0.8525	0.8495	

## Data Availability

The Python scripts and some data files are openly available in Zenodo at https://doi.org/10.5281/zenodo.18067784.

## Supplementary Materials

The following are available online at https://www.mdpi.com/article/10.3390/diagnostics16020310/s1, Figure S1: GUI for corneal ectasia score prediction. Abbreviation: GUI = Graphical User Interface; Figure S2: File selection window for choosing a 3dv file; Figure S3: Extracted and scaled images from the selected 3dv file; Figure S4: Eye selection window; Figure S5: Predicted corneal ectasia score and diagnosis.

## Abbreviations

The following abbreviations are used in this manuscript:

AI	Artificial Intelligence
CNN	Convolutional Neural Network
CSV	Comma-Separated Value
FP	False Positive
FN	False Negative
Grad-CAM	Gradient-Weighted Class Activation Mapping
GUI	Graphical User Interface
MAE	Mean Absolute Error
MSE	Mean Squared Error
OCT	Optical Coherence Tomography
PNG	Portable Network Graphic
PPV	Positive Predictive Value
RAM	Random-Access Memory
ROC	Receiver Operating Characteristic
TP	True Positive
TN	True Negative
