# Supplementary figures and images for: A Deep Learning-Based Graphical User Interface for Predicting Corneal Ectasia Scores from Raw Optical Coherence Tomography Data
Source: Diagnostics (Basel). 2026 Jan 18;16(2):310. doi: 10.3390/diagnostics16020310 (PMC12839634; doi:10.3390/diagnostics16020310)

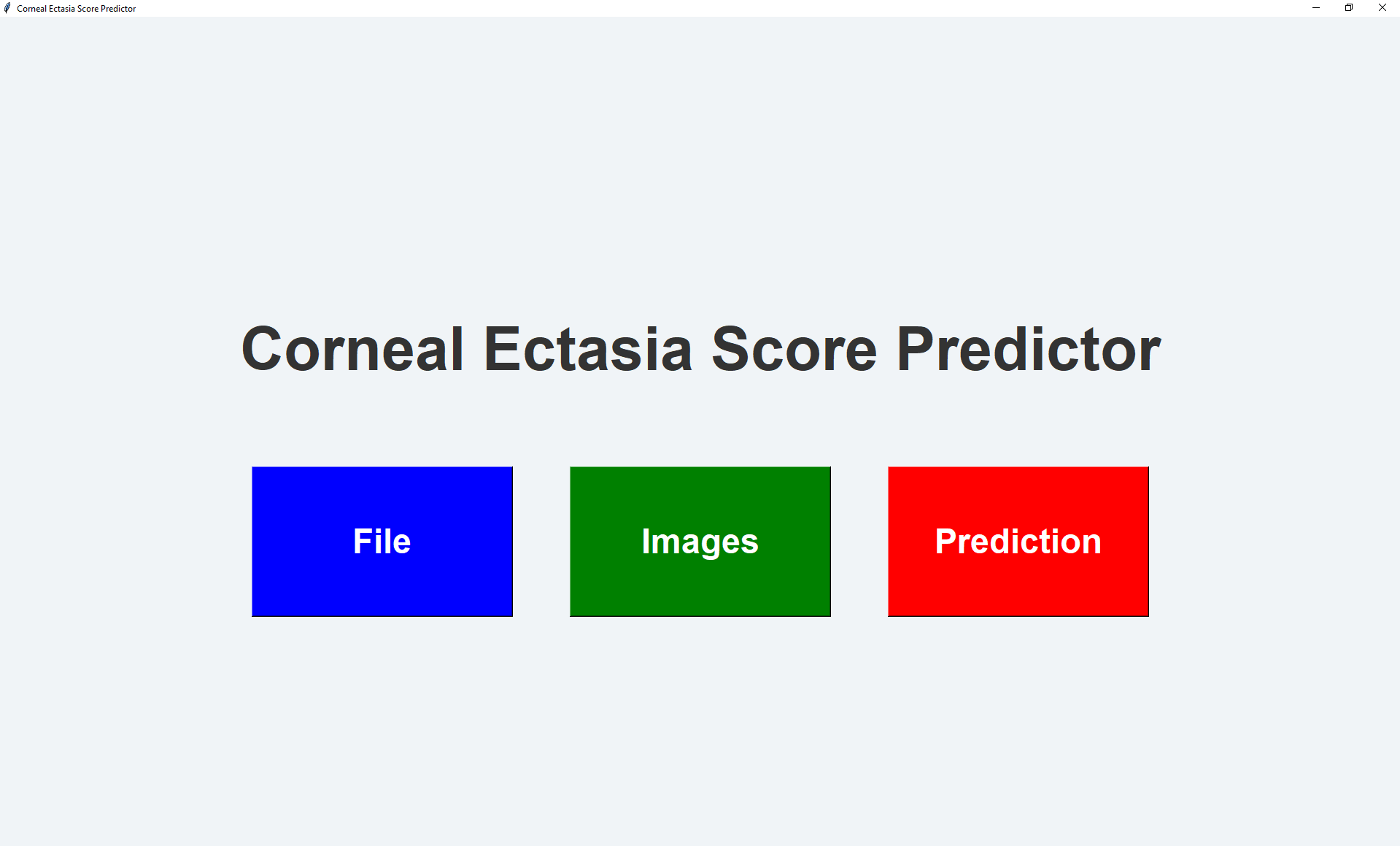

Supplement: Supplementary file 1 [file diagnostics-16-00310-s001.zip › Figure S1.tif]

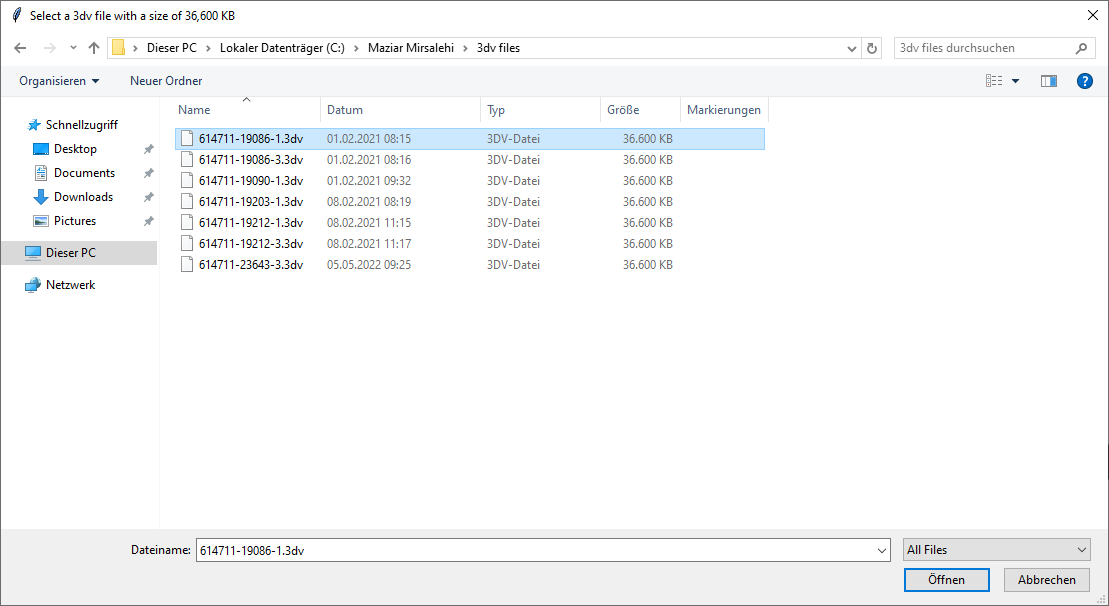

Supplement: Supplementary file 1 [file diagnostics-16-00310-s001.zip › Figure S2.tif]

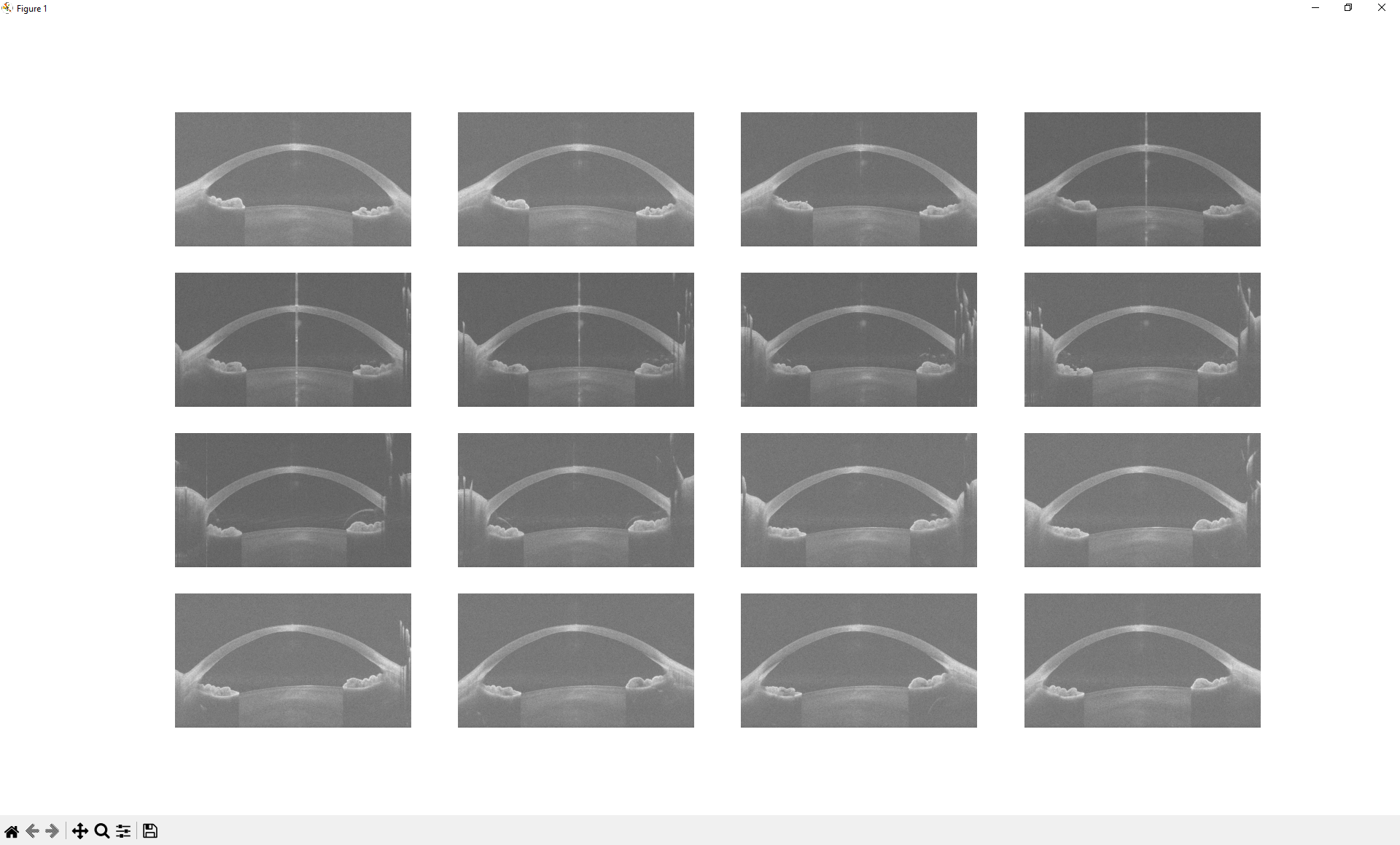

Supplement: Supplementary file 1 [file diagnostics-16-00310-s001.zip › Figure S3.tif]

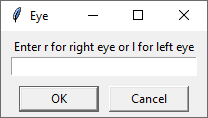

Supplement: Supplementary file 1 [file diagnostics-16-00310-s001.zip › Figure S4.tif]

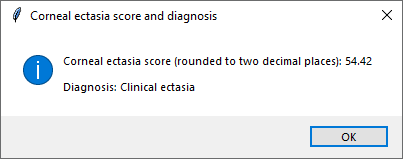

Supplement: Supplementary file 1 [file diagnostics-16-00310-s001.zip › Figure S5.tif]
